# Advances in Graves' Disease

**DOI:** 10.1155/2012/809231

**Published:** 2012-05-20

**Authors:** Juan C. Galofré, Leonidas H. Duntas, L. D. Premawardhana, Terry F. Davies

**Affiliations:** ^1^Department of Endocrinology and Nutrition, Clinica Universidad de Navarra, 36 Pio XII Avenue, 31080 Pamplona, Spain; ^2^Endocrine Unit, Evgenidion-Hospital, University of Athens, 11528 Athens, Greece; ^3^Department of Medicine, C2 Link, University Hospital of Wales, Cardiff CF14 4XN, UK; ^4^Division of Endocrinology and Metabolism, Mount Sinai School of Medicine, James J. Peters VA Medical Center, New York, NY 10468, USA

 “*From the very commencement the student should set out to witness the progress and effects of sickness and ought to persevere in the daily observation of disease during the whole period of his studies*.”

 It was Dr. Robert J. Graves who used to pronounce this statement at the inauguration of his yearly university lectures in Dublin. This was in the nineteen century and he had only just described Graves' disease, the most common hyperthyroid condition that is so widely recognized today. There could be at least two complementary ways of interpreting Dr. Graves' perennial advice. The first way is from a *practical* viewpoint. It emphasizes the importance of observation and monitoring clinical evolution. This is an important good-practice guide for doctors (and students) that are at a patient's bedside. This *practical* approach promotes a deep scrutiny of the disease, taking into consideration that it is not an abstract concept, but an ailment embodied in a given patient. The second interpretation of Graves' statement could be more *theoretical*. Thus the significance of the statement supports the concept of clinical and laboratory research. Physicians must participate in research at all levels: basic, translational, and clinical. Dr. Graves' counsel encourages efforts to achieve a deep knowledge of disease as individual entities. Therefore, both objectives, practical and theoretical, are closely entwined—laboratory advances connected to the bedside—what we now call translational medicine. Unfortunately, this link is often weak.

 Since 1835, when Graves described his disease, dramatic progress has been made in our knowledge of the illness. During these nearly two centuries, we have come to understand a variety of molecular, genetic, and autoimmune mechanisms that give rise to and maintain the disease. However, it is also true that despite recent advances, the clinical management of Graves' disease has changed very little over the last few decades. Nevertheless, thanks to a group of outstanding physician-investigators able to integrate the laboratory with the bedside, we sense that exciting changes in the management of Graves' disease are at hand. Currently, for instance, there are several molecular target therapies under development that will significantly alter the clinical management of the disease within the next few years. This special issue is intended to highlight some of the most recent breakthroughs in this area. The issue includes a complete overview: from basic reviews to clinical papers through translational studies.

T. F. Davies et al. summarizes the new genetic insights into autoimmune thyroid diseases (AITDs), a complex topic that is actively being investigated. At present, more than twenty genes have been associated with AITD that can be categorized into two groups: immune regulatory genes (which are common to other autoimmune diseases) and thyroid-specific genes. Despite the described gene-AITD association, the individual gene contribution to AITD development is complex. Furthermore, no single polymorphism seems to contribute substantially to the development of the autoimmune reaction in thyroid diseases. The emerging evidence indicates that some environmental and/or epigenetic modifications over a predisposing genetic background could change individual gene expression, which subsequently elicits AITD manifestation. Although new genetic findings have emphasized the identification of the environmental components that interact with host genetic factors in other autoimmune diseases, this approach has been elusive so far for AITD. Unfortunately for the clinician, the genetic profiling of AITD patients is unlikely to be productive in the near future, with the corresponding limitation in the development of new strategies in prevention and predictive treatment.

 The role of microchimerism in Graves' disease is the subject of J. C. Galofré's review article. In this paper the author updates and reviews the main evidence that suggests a close relationship linking fetal microchimerism and the development of AITD. Certainly, the presence of intrathyroidal fetal cells within the maternal thyroid is an attractive candidate mechanism for the modulation of Graves' disease in pregnancy and the postpartum period. At present, however, microchimerism responsibility in the generation of AITD remains a hypothesis.

 In their review articles, M. Žarković and L. H. Duntas address an important and emerging matter: the role of oxidative stress on the pathogenesis of Graves' disease and its specific treatment, respectively. M. Žarković describes how oxidative stress is indeed an environmental factor that induces and maintains the development of Graves' ophthalmopathy. Subsequently L. H. Duntas reviews the emerging role of selenium in the treatment of Graves' disease and ophthalmopathy. Both contributors tackle the question of the inflammatory process in AITD. The imbalance of the antioxidant-oxidant mechanism is described in detail. The authors illustrate how there is an increased production of radical oxygen species and cytokines, which sustain the autoimmune process and perpetuate the disease. It is stressed that selenium, a potent antioxidant, has been recently applied in patients with mild Graves' ophthalmopathy, slowing the progression of disease, decreasing the clinical activity score, and appreciably improving the quality of life. Questions remain open to further research such as whether enforced selenium nutritional supplementation has the same results on Graves' disease and whether prolonging selenium administration may have an impact on the prevention of disease.

 S. El-Kaissi and J. R. Wall contribute with an original research article. The authors study the determinants of extraocular muscle volume (assessed by MRI) in 39 patients with Graves' disease. The study shows that patients with recently diagnosed Graves' disease and extraocular muscle volume enlargement have higher serum TSH and more severe hyperthyroidism at baseline than patients without extraocular muscle enlargement, with no difference in anti-TSH-R antibody positivity when comparing both groups.

C. Kamath et al. summarize the role of thyrotrophin receptor antibody (TR-Ab) assays in Graves' disease. TR-Ab assays commonly used and widely available to clinicians, measure thyroid-binding inhibiting immunoglobulins (TBII or receptor assays), and do not differentiate between stimulating (TRS-Ab), neutral, and blocking antibodies (TRB-Ab). This limitation can induce confusion in managing Graves' disease patients although the patient may be the best bioassay. The current 2nd-3rd generation receptor assays are highly sensitive and specific when used to differentiate between the functional types of TR-Ab. The authors also encourage measuring TR-Ab in pregnant women under appropriate circumstances. Unfortunately, current data are not conclusive about its use in predicting the outcome of Graves' disease after antithyroid drug therapy, as there is a significant variability in assay methodology, population characteristics (e.g., their iodine intake), and study design in published data.

 An example of the inherent difficulties in interpretation of positive TR-Ab significance, as postulated in the C. Kamath et al. review article, is illustrated by N. Takasu and M. Matsushita original research article. The authors study the changes in serum TRB-Ab and TRS-Ab levels in 34 TRB-Ab-positive patients with hypothyroidism and in 98 TRS-Ab-positive Graves' patients with hyperthyroidism. The study covers a ten-year period. Serum TRB-Ab levels remained elevated during the entire study period in half of the patients with initial hypothyroidism. Interestingly, hypothyroid patients were divided according to the presence of atrophic or goitrous autoimmune thyroiditis. Despite the presence of positive TRB-Ab, all the patients with the goitrous form recovered from hypothyroidism whereas only 21% of the atrophic patients evolved to euthyroidism. Around 10% of the positive TRS-Ab patients remained with elevated circulating TRS-Ab levels at the end of the follow-up and these patients continued to have hyperthyroidism due to Graves' disease. On the other hand, remission of Graves' disease occurs in 82% of patients in whom TRS-Ab disappeared from the serum. The switch from TRB-Ab to TRS-Ab or vice versa took place in 5.8% and 2.0%, respectively, always inducing a change in the gland function. The authors' main conclusion is that positive TR-Ab may be associated with two manifestations: hyperthyroidism and hypothyroidism.

 M. O. Hegazi and S. Ahmed review article focuses on atypical clinical manifestations of Graves' disease. Some of the atypical features are specifically related to Graves' disease (including anemia, vomiting, jaundice, and right heart failure), while others are also similarly found in patients with other forms of hyperthyroidism. Pulmonary hypertension is reported to be associated with Graves' disease and reportedly responds to its treatment. Such atypical signs and symptoms should be considered suspect and should not be allowed to delay diagnosis or unnecessary investigation.

 We sincerely hope that the present volume will help clinicians who work in the stimulating field of thyroidology *to persevere in the daily observation of disease during the whole period of their studies *for the benefit of their patients.

*Juan C. Galofré* *Juan C. Galofré* *Leonidas H. Duntas* *Leonidas H. Duntas* *L. D. Premawardhana* *L. D. Premawardhana* *Terry F. Davies* *Terry F. Davies*

